# Evaluating Current Molecular Techniques and Evidence in Assessing Microbiome in Placenta-Related Health and Disorders in Pregnancy

**DOI:** 10.3390/biom13060911

**Published:** 2023-05-30

**Authors:** Aleksandra Stupak, Wojciech Kwaśniewski

**Affiliations:** 1 Department of Obstetrics and Pathology of Pregnancy, Medical University of Lublin, Staszica Str. 16, 20-081 Lublin, Poland; 2 Department of Gynecological Oncology and Gynecology, Medical University of Lublin, 20-081 Lublin, Poland

**Keywords:** microbiome, genomics, pregnancy, placenta, fetal growth restriction, 16S DNA sequencing, metagenomics sequencing

## Abstract

The microbiome is of great interest due to its potential influence on the occurrence and treatment of some human illnesses. It may be regarded as disruptions to the delicate equilibrium that humans ordinarily maintain with their microorganisms or the microbiota in their environment. The focus of this review is on the methodologies and current understanding of the functional microbiome in pregnancy outcomes. We present how novel techniques bring new insights to the contemporary field of maternal–fetal medicine with a critical analysis. The maternal microbiome in late pregnancy has been extensively studied, although data on maternal microbial changes during the first trimester are rare. Research has demonstrated that, in healthy pregnancies, the origin of the placental microbiota is oral (gut) rather than vaginal. Implantation, placental development, and maternal adaptation to pregnancy are complex processes in which fetal and maternal cells interact. Microbiome dysbiosis or microbial metabolites are rising as potential moderators of antenatal illnesses related to the placenta, such as fetal growth restriction, preeclampsia, and others, including gestational diabetes and preterm deliveries. However, because of the presence of antimicrobial components, it is likely that the bacteria identified in placental tissue are (fragments of) bacteria that have been destroyed by the placenta’s immune cells. Using genomic techniques (metagenomics, metatranscriptomics, and metaproteomics), it may be possible to predict some properties of a microorganism’s genome and the biochemical (epigenetic DNA modification) and physical components of the placenta as its environment. Despite the results described in this review, this subject needs further research on some major and crucial aspects. The phases of an in utero translocation of the maternal gut microbiota to the fetus should be explored. With a predictive knowledge of the impacts of the disturbance on microbial communities that influence human health and the environment, genomics may hold the answer to the development of novel therapies for the health of pregnant women.

## 1. Introduction

Microorganisms have played a crucial role in the creation of fertile land that sustains crops and livestock and, therefore, human populations. Even within our own bodies, bacteria are required for digestion and immune system development. Since its inception, the study of microorganisms has relied on technological advancements. In 1995, the first description of a full genome sequence of an autonomous life form propelled “genomics” to prominence [1,2]. The result of dynamic interactions within a community at the genomic, metabolic, and structural levels is a network of microbial species whose collective activities are frequently greater than the sum of their individual functions. This is because the network is more complex than the individual species. Some microbial communities, for instance, are capable of degrading harmful substances in a way that individual species cannot [3]. The genomic technologies are now sufficiently developed to decode the genetic properties of increasingly complex microbial communities [4].

The microbiome refers to the collection of all the microorganisms (bacteria, fungi, and virus) and their genomes in the environment. The microbiota, on the other hand, usually refers to the microorganisms’ genomes. The microbiome consists of all saprophytic microorganisms, commensals, and parasites that inhabit human, animal, plant, and soil species, as well as their genomes and interactions [5]. The microbiome is not randomly distributed throughout the body but rather resides in specific locations, including the skin, respiratory tract, digestive tract, and reproductive organs. The unique population of microorganisms corresponds closely not only to the body locations of individuals of a certain species but also to their ages (newborns or adults).

The Human Microbiome Project (HMP) has discovered that bacteria inhabiting environments such as the stomach, skin, and vagina vary significantly among healthy individuals [6]. After quality control, 4788 specimens from 242 screened and phenotyped individuals (129 males and 113 females) were utilized for 16S rRNA gene analysis through 454 pyrosequencing, and 681 samples were sequenced using paired-end Illumina shotgun metagenomic reads. It was determined that the median alpha diversity of operational taxonomic units OTUs (number of different microorganisms) was greatest in the saliva; however, the median beta diversity was among the lowest (the population shared between similar organisms). The beta diversity of the skin samples was greater than the alpha diversity. Due to the presence of different *Lactobacillus* spp., the vagina showed the lowest alpha diversity and very low beta diversity at the genus level but a very high diversity across OTUs. No microbe was found to be uniformly prevalent across all body habitats or people in this investigation. Within and across bodily habitats, microorganisms were individualized, functionally relevant, and influenced by physical conditions (oxygen, pH, and humidity), immunological effect, and microbial competition. 

Despite the variances between individuals and animal species, some bacterial species are always present in the microbiomes of specific body regions (the skin, intestinal contents, and genitourinary system). The gut has the largest and most diversified microbiome in humans and animals. However, regardless of individual variation, the gut microbiome always contains bacteria from distinct genera: *Faecalibacterium*, *Ruminococcus*, *Eubacterium*, *Dorea*, *Bacteroides*, *Alistipes*, and *Difidobacterium* [7].

Intestinal bacteria have a wide range of activities, including the ability to affect the immune system. They stimulate both specific and nonspecific immunological processes in the organism, impacting the lymphoid tissue linked with the mucosa (MALT). They induce the production of IgG, IgM, IgA, SIgA, and SIgM antibodies, which inhibit adherence to the intestinal mucosa epithelium and penetration into the mucosal membranes. Saprophytic bacteria of the digestive system suppress inflammation and impact the intestinal barrier’s sealing. In infants with allergies, *Lactobacillus acidophilus* and *L. rhamnosus* repair the integrity of intercellular connections of the intestinal epithelium disrupted by TNF and INF [8]. Second in terms of the number of bacteria, the skin microbiome not only guards against diseases but also triggers an immune response by influencing the skin’s T cells [9,10]. Many ailments, such as inflammatory bowel disease, periodontal disease, “bacterial vaginosis”, and the overgrowth syndromes associated with the use of antibiotics, are examples of conditions that may be brought on or made worse by changes in the population of microorganisms [11]. Alterations to the microbiota that are brought into contact with the human body have the potential to bring about disease. The anticipation of the impact of these changes on human tissues would allow for more precise management or prevention of some of those diseases [12].

Today, genomic technologies are transforming microbiology. The greatest way to enhance the research into microbial genomes is by adopting diverse methodologies and tackling numerous degrees of complexity in study systems. The metabolic and functional pathways (protein families) of the complete human microbiome are being recognized using genetic and metagenomic data. Future research on metatranscriptomes and metaproteomes will establish a connection between the microbiome and human health. This paper discusses recent research and evidence about assessing the microbiome in placenta-related health and disorders in pregnancy.

## 2. Pregnancy

### 2.1. Physiology of Pregnancy and Microbiome

There is evidence that bacteria have a role in modifying physiological activities related to human pregnancy, such as increased weight gain and insulin resistance, which may be traced throughout the trimesters [13]. Throughout pregnancy, the gut microbiome undergoes major changes. During the advance of pregnancy, the abundance of certain phyla (Proteobacteria and Actinobacteria) increases, but total species diversity declines, and interindividual variability increases [14]. The makeup of the maternal microflora may undergo adaptive changes during pregnancy. For instance, a higher quantity of estrogen increases glycogen formation in the vagina, which, in turn, changes the vaginal microbiota dynamics [15]. During pregnancy, progesterone may affect the microbial structure of the gut, such as by increasing the abundance of *Bifidobacterium* [16]. Yet immunological modifications also affect the microbial makeup; for example, an increase in IL-15 might decrease the number of butyrate-producing bacteria [17]. Adaptations of the maternal gut, vaginal, and oral microbiome during different times of healthy pregnancies are distinguishable due to changes in the microenvironment of different body locations and features during the several trimesters [18]. The modifications in the microorganisms that occur during a healthy pregnancy promote adaptation to the physiological circumstances of pregnancy, which are notably distinct from disease states.

The maternal microbiome in late pregnancy has been widely investigated (Table 1). Data on maternal microbial alterations in the first trimester are rare. The intestinal microbiota in the first trimester is comparable to the microbiota in nonpregnant women [19]. At mid-pregnancy, the most common phyla in the microbial community structure are as mentioned in Table 1, while the abundance of *Bifidobacteriaceae* and *Enterobacteriaceae* is considerably elevated [20]. During the third trimester, maternal gut microbes experience the most remarkable changes, including decreases in alpha diversity and butyrate-producing bacteria with anti-inflammatory effects but increases in the beta diversity of the microbes listed in Table 1.

These vaginal microbial alterations during pregnancy may be partially attributable to estrogen-induced increases in glycogen levels [18]. Nevertheless, DiGiulio found that the makeup and diversity of the vaginal microbiome remain rather consistent throughout pregnancy [21]. Another study discovered that the makeup of the vaginal microbiome in late pregnancy is similar to that of the nonpregnant state [22].

### 2.2. Microbiome and Maternal Immune System

**Figure 1:**
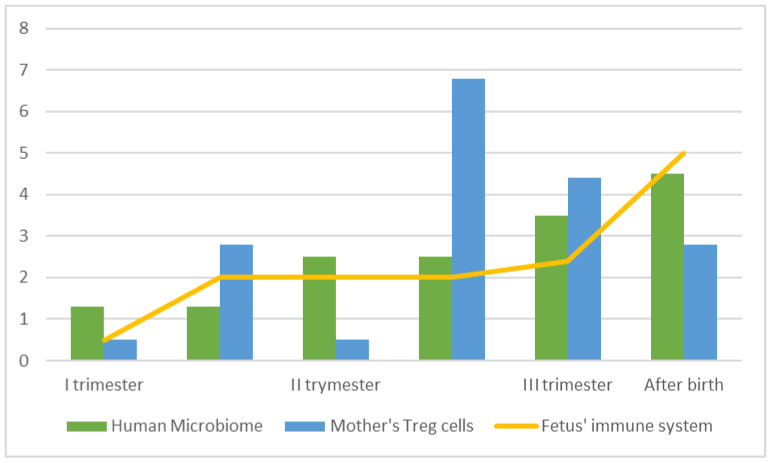
Illustration of the association of changes in the human microbiome with the maternal and fetal immune systems [24,25,26].

Several prospective advantages are associated with a placental microbiome and the mechanisms that occur at this interface [23]. The bacteria can be transported via the placenta and have a role in early immune development; however, the efficiency or benefit of this transfer is dependent on certain microorganisms that may be associated with newborn or early childhood immunological disorders [24]. If maternal antigens help in the transmission of microorganisms and then boost the establishment of early immune cell populations, the decreased maternal immunity may impact the infant’s immunological development (see Figure 1). The illustrative figure approximates probable fluctuations in maternal Treg cells and the presence of the placental microbiome that contribute to the adaptation and maturation of the fetal immune system, which continues after birth [25,26]. In line with this thesis, in prenatal trials with mice, antibiotics induced dysbiosis, impaired early immunological development, and increased susceptibility to viral infections [27].

The intestinal epithelium is a physiological barrier with macrophages, dendritic cells, mast cells, and transmembrane proteins, e.g., Toll-like receptors (TLRs). The microbiome exerts its influence via modulating the expression of TLRs, inflammasomes, C-type lectins, RIGs, and helicases with similar functions [28,29]. Toll-like receptors are predominantly located on the surface of immune system cells and, to a lesser degree, on epithelial cells of the respiratory and gastrointestinal tracts, adipohemocytes, fibroblasts, and cardiomyocytes. TRL-2 and TRL-4 receptors emerge on the surface of enterocytes between 18 and 21 days of fetal life and identify pathogen recognition receptors (PRRs), such as endotoxin (LPS), peptidoglycan, glycoproteins, uronic acids of bacteria, and heat-shock proteins. Genes governing the immune response are expressed as a result of the activation of particular signaling pathways. Several proinflammatory cytokines (IL-1, IL-6, IL-8, IL-12, and TNF) are induced. One PRR may distinguish many molecular patterns. PRRs are recognized as part of the normal immune response and can begin the onset of a systemic inflammatory response [30].

Commensals and skin pathogens regulate the production of antibacterial peptides in distinct ways, activating distinct signaling pathways. Through activating Toll-like receptor 7 (TRL-7), EGFR, and the transcription factor NF-κB, commensal cutaneous staphylococci generate defensins (HBD-3) and RNAse 7 in keratinocytes. Commensals augment keratinocytes’ intrinsic resistance to infections by boosting adenosine monophosphate (AMP) expression and reducing NF-κB repression. In contrast, pathogenic staphylococci stimulate the mitogen-activated protein kinase and phosphatidylinositol-3 kinase signaling pathways and inhibit NF-κB [31].

Respiratory commensals have an effect on CD4^+^ and CD8^+^ T lymphocytes [32]. *Bifidobacterium infantis*, a component of the intestinal microbiome, exemplifies the effect of the intestinal microbiome on the neuroendocrine system by altering the metabolism of tryptophan, subsequently influencing the generation of serotonin [33].

### 2.3. Human Placenta

Placental membranes include cells that destroy bacteria and actively protect the fetal environment. Some examples of these cells are trophoblasts, natural killer cells, leukocytes, and macrophages. Placental membranes also contain other immune cells called leukocytes [34]. Bacterial cells may cross the placental barrier in the form of bacterial ligands; this process is assisted by maternal antibodies, which contribute to the formation of infant immunity [24]. Nevertheless, as placental tissue contains antimicrobial components, it is likely that the bacteria that are detected in placental tissue are not live bacteria but rather fragments of bacteria (e.g., DNA) that have been destroyed by the immune cells of the placenta. This is the most likely explanation. However, some microbes may be covertly present inside trophoblast cells, which would make identification even more challenging. Recent research carried out by Parnell convincingly showed the possibility of symbiosis among different species of *Ralstonia* [35]. *Ralstonia insidiosa* was not only found by FISH in placental biopsies but was also able to elude inflammation and cell death while replicating in the intracellular vacuoles of trophoblasts ex vivo. Additionally, the presence of *Ralstonia insidiosa* in the placenta did not induce preterm birth in a mouse model of the condition. The fact that this study provided a mechanistic understanding of the probable function of placental bacteria makes it a very important body of research. However, it is necessary to note that the placenta may have diverse impacts depending on the physical region it occupies in the body. A few investigations demonstrated variations in the microbial load between the maternal and fetal sides of the placenta, with the maternal side typically yielding higher log copy counts. This is because the maternal side of the placenta is closer to the mother [35,36,37]. Along the same lines as the placenta, it was formerly thought that the uterus was an infertile organ; however, current research, as well as a comprehensive study [38], has started to change this point of view. The probability that germs have invaded the uterus significantly increases the likelihood that the same infectious agents will be passed on to the child.

### 2.4. Healthy Placental Microbiome

Currently, the fetal immune system is believed to be substituted by maternal antibodies transferred through the placenta. The current hypotheses suggest that microorganisms, including endotoxins and bacterial DNA fragments, might possibly stimulate the immune response [23]. Two theoretical pathways are suggested to describe the way in which the gestational intestinal microbiome could affect the fetus [39]. Microbes at the maternal site are believed to enter the bloodstream from the intestinal epithelium and reach the placenta. This is thought to be associated with proinflammatory changes (increased serum endotoxin and zonulin concentrations, as well as increased fecal calprotectin levels) in the bacterial environment, enhancing mucosal permeability and microbiota translocation through maternal circulation probably to fetal gut colonization. The maternal gut microbiome indirectly regulates fetal growth and development through a second mechanism. The microbiome synthesizes these soluble factors or metabolizes chemicals from the intestinal lumen. Maternal colonization alters the gene expression profile of the fetus’s gut epithelium encoding homeostasis, integrity, and differentiation [24]. The levels of 5-aminovaleric acid betaine, trimethylamine *N*-oxide, catechol-*O*-sulfate, hippuric and pipecolic acid, acetic acid, propionic acid, and butyric and caproic acid are the main soluble end-products of bacterial metabolism and may control the balance of immune responses [40,41]. Other studies have demonstrated that, in addition to live bacteria, endogenous microbial compounds (e.g., flagellin, tryptophan, or lipopolysaccharide) can probably influence fetal tissues [42]. Thus, maternal gut microbiota compounds can reach fetal compartments and stimulate the fetal immune system. Although these discussions still do not conclusively support the presence of bacteria in the placenta, they try to explain their potential role in the development of the fetal immune system and open the field for research on changes in the placental microbiome in pathological pregnancies.

Most of the research demonstrates that the genesis of the placental microbiome in healthy pregnancies is oral and not vaginal. Two of the twenty-four investigations found no microbes in placental samples, with one study analyzing only eukaryotic microbiota (fungi) [43,44]. *Firmicutes* was the most frequently identified bacterial phylum in placental tissue samples, with 18 studies (75%), followed by the others mentioned in Table 1, along with others, such as *Cyanobacteria*, *Spirochaetes*, and *Acidobacteria* [23]. In Aagaard’s study, the human placental microbial community was found to be dominated by the major phylum *Proteobacteria* [45]. Microbiota research also found physiological bacteria in the placenta, amniotic fluid, umbilical cord, fetal intestine, and meconium [39].

As the microbiota of neonates varies depending on the manner of delivery, this has also been predicted for placenta samples from vaginal and cesarean section births [46]. There are studies, however, that did not find variations in the microbiota based on the delivery modality, and, when differences were observed, they were likely due to contamination [47,48,49,50,51]. The findings should, thus, be presented individually per delivery mechanism. The most secure way to retrieve placental tissue is via elective cesarean sections, a sterile surgery with intact membranes. Antibiotics are usually administered before the procedure, which may change the culture results. In healthy pregnancies, the current studies cannot rule out the presence of a placental microbiome with low biomass. Some of the germs found in the placenta may have been ingested, identifying a high likelihood that the core microorganisms were significantly influenced by contamination; thus, further research is necessary to establish the microbiome variations in healthy people. Furthermore, studies analyzing the effect of these microorganisms on the development of the human immune system are essential for the progress of this study.

## 3. Placenta-Related Syndromes

Placental disorders include pregnancy loss, fetal growth restriction, preeclampsia, preterm delivery, premature rupture of membranes, placental abruption, and intrauterine fetal death [52]. The two-stage process of implantation and placental development involves maternal–fetal interaction and adaptation [53,54]. The changes in the syncytiotrophoblast, trophoblast, and uterine arteries are under the influence of uterine natural killer cells and regulatory T cells [55,56]. Angiogenic factors, such as the PlGF (placental growth factor) and sFLT (soluble fms-like tyrosine kinase-1) regulate placental angioneogenesis, vasculogenesis, and maternal circulatory adaption [57]. Maternal immunotolerance, uterine natural killer, or regulatory T-cell dysregulation in placental disorders can cause syncytiotrophoblast and trophoblast ischemia, hypoxia, and angiogenic factor imbalance. A placental syndrome develops on the basis of impairment onset, intensity, and association with other dysfunctions, e.g., dysbiosis. Our research is focused on alterations in the diversity and structure of the microbiome in preeclampsia and FGR, as important problems in obstetrics, affecting 5–10% of pregnancies worldwide [58,59]. However, the incidence of FGR in women with severe preeclampsia can exceed 22% [60].

## 4. Methodology of Placental Microbiome Assessment

Among the obstacles we face, the methodologies, implementation, and viewpoints of genomics that may be applied most effectively to comprehend microbial communities as a whole must be established. Genomics and other molecular approaches are incredibly effective and alleviate many of the issues associated with cultivation-based procedures. Various “omics” approaches have been used in the studies of the placental microbiome [61]. Next-generation sequencing (NGS) has enabled genomic techniques to better comprehend the complicated microbial environment from different biological samples. Metagenomics mostly covers microbial ecosystem taxonomy and function; however, it cannot answer all biological questions. The main constraints are different lab NGS systems and bioinformatics tools. Metatranscriptomics (RNAseq) allows whole genome analysis of the active microbial community and biological markers expressed in the human microbiome. Nevertheless, metatranscriptome data analysis uses metagenomics-like bioinformatics methods. The culture-based omics method, culturomics, uses diverse culture conditions, MALDI-TOF mass spectrometry, and 16S rRNA sequencing to quickly identify bacterial species [62]. Another method for analyzing the content of the microbial communities is the examination of the proteins, so-called “metaproteomics” [63]. Metaproteomic analysis consists of four stages: extraction and purification of proteins, enzymatic digestion of proteins into peptides, separation of peptides (usually by chromatography, followed by mass spectrometric analysis), and protein identification through database sequence comparison. The limitations of this method are that many peptides are common to many bacterial species, as well as the lack of reliable bioinformatics databases [61].

In the study of Aagaard et al., similar to other earlier microbiome profiles produced from low-biomass tissue samples, placental metagenomic datasets contained a substantial quantity of human sequencing (>99%) [45]. Therefore, the initial step in processing WGS reads was to eliminate human contamination and verify the dataset’s integrity and confidentiality. There were a few limitations of this study, one of which was the method itself. For that, the authors used a large number of primary specimens with a sterile clean protocol to receive reliable data. However, 16S RNA analysis is more sensitive than metagenomics and should not be instantly discounted as a less sophisticated technique [64].

In five studies, the types of nonselective anaerobic and facultative aerobic cultures for bacterial isolation ranged in number from one to seven [65,66,67,68,69]. In a review of Zakis’s research, which used 16S rRNA gene sequencing [23,42,43,45,49,50,65,66,70,71,72,73,74,75,76,77,78], in situ immunofluorescence detected *Porphyromonas gingivalis* antigens in placental specimens [79]. Metagenomics sequencing was conducted in five investigations [45,50,66,67,74]. Using nested PCR, bacterial DNA was amplified in two investigations [50,66]. A number of studies targeted certain microorganisms using species-specific PCR [48,50,52,67,68,69]. The researchers found 2–19 urogenital pathogens and periodontal pathogens, as well as the sip gene encoding the *Streptococcus agalactiae* immunogenic surface protein (sip) [48,50,67,69,73]. Three investigations stained placental samples using distinct histology techniques [65,67,68].

Among the research projects deemed to be of sufficient quality, several 16S rRNA gene hypervariable (V) regions were sequenced. However, 16S rRNA sequencing has been applied for decades for bacterial phylogenetic studies, oftentimes suffering from low resolution. These regions had different sites for PCR. One of the research projects taken into consideration assessed numerous V-regions and found that, in the majority of samples, the signal was distinct from the controls due to variations in the V4 region of the bacterial DNA [49]. Several primers either amplified the controls by a considerable amount or did not amplify anything at all. The usage of V3–V4 was supported by the results of two separate studies, one of which was conducted on the skin, with the other conducted on the oral microbiota [80,81]. In more recent studies on placental samples, positive results were reported when primers targeting the V3–V4 region were used but not when the same primers as those used for other V4 samples were used [66,82]. Better detection rates were seen when the enrichment technique was used prior to the sequencing of the V4 region [83]. More studies are planned using this methodology for placental tissues. Other researchers have stated the use of V1–V2 primers, which may allow environmental contamination, as a potential reason for their inability to discover microbes in their tissues when compared to the controls. This was the case when those researchers were unable to find microbes in their own samples [36,47,49]. Fuks et al. proposed a fundamentally new technique for low-biomass samples called the Short Multiple Regions Framework (SMURF) [84]. This was accomplished by amplifying the sample using a number of different variable locations. By combining the data and the findings from many different locations, an enhanced resolution of up to 100 times for a fictitious town can be achieved. Comparisons across studies need to be conducted with extreme caution because of the wide variety of research approaches used and the disparity in the sizes of the samples. Subsequent studies that used NextGen techniques provided more evidence of the diversity and complexity of the vaginal microbiota seen in healthy women. These microbiota are primarily classified into five different community state types (CSTs) on the basis of the bacteria that predominate in each type [18]. Studies that made use of 16S metagenomics approaches showed that the composition of the oral microbiota does not change over the course of pregnancy [21].

Proteomics enables the analysis of microbial protein expression. Metaproteomics uses mass spectrometry to discover protein patterns in complex samples, such as the gut microbiota. The latest mass spectrometry technology allows large-scale microbial protein characterization in the placenta. Despite technology advancements, bioinformatics analysis remains a challenge. In the study of Stupak et al., proteomics (LC–ESI-MS/MS mass spectrometry) and bioinformatics studies of placental biomass were used [85]. MASCOT 2.4.1 (Matrix Science, London, UK) was used to search the Uniprot 2019_02 (561,356 sequences; 201,858,328 residues) database with bacterial sequences and a filter to examine the spectrum data. Acceptable protein identification required the identification of at least two peptide fragments per protein. The Exponentially Modified Protein Abundance Index (emPAI) was applied to perform a nonlabel quantitative comparison of proteins across the studied samples. Through an analysis of the proteinogram in the material collected from the study group (*n* = 18), 145 bacterial proteins were detected. In the control group, there were 628 bacterial proteins. According to the proteomic data, there were also bacteria identified in both groups. This method highlighted the placental dysbiosis in FGR pregnancies.

Unfortunately, none of these methodologies differentiate among living bacteria, dead microorganisms, and bacterial fragments (DNA, proteins, or metabolites) as a response to the immune system. Therefore, they are all sources of positive “bacteria” signals. Over the last 10 years, controversies have accumulated in the placental microbial community [34,45,50]. A 2019 publication in *Nature* dispelled the doubts and refuted the results of previous authors [45,50]. The study identified different patterns of contamination (1) of the placenta with real bacteria during the process of labor and delivery, (2) of the biopsy when it was washed with PBS, (3) of the DNA during the extraction process, (4) of the reagents used to amplify the DNA prior to sequencing, and (5) from the reagents or equipment used for sequencing. In the opinion of Young, simply demonstrating that we can detect microbes using culture-independent methods (e.g., 16S rRNA or shotgun metagenomics or FISH) is not enough [64]. We expect this community to be stable over time, reproduce in the placenta, and remain metabolically active. However, the aim of the research on dysbiosis in placenta-related syndromes is not about proving the presence or absence of microbial DNA but analyzing the potential role of this “colonization” as it stimulates and influences the pathomechanism of different pathologies of pregnancy. The same opinion was presented by Perez in 2017, i.e., that future studies should emphasize the postnatal acquisition of the gut microbiome and its importance to health, as well as the possible role of the prenatal exposure of the fetus to microbial metabolites and compounds that originate from the maternal gut microbiota [34].

All methods used for the studies on the placental microbiome are listed in Table 2.

## 5. The Influence of the Method of Delivery on the Results

Concerns have been raised about the interpretation of data indicating in utero fetal exposure to the maternal microbiota due to the increasing number of studies conducted. In all microbiome investigations, different methods of delivery were used: vaginal delivery (VD), spontaneous cesarean section (sp CS), and elective cesarean section (el CS). As the microbiota of neonates varies according to the mode of delivery, this has also been predicted for placenta samples from vaginally born and cesarean section patients [46]. However, there are studies that did not discover variations in microbiota according to the manner of delivery, and, when differences were observed, they were most likely due to contamination [47,48,49,50,51]. The findings should, thus, be presented individually per delivery mechanism. The safest placental tissue may be obtained through CS, a sterile procedure in which the amniotic membranes are still intact. However, according to some researchers, the antibiotics administered before the surgery may alter the culture findings. Several authors have provided a compelling case for the absence of external contamination during the collection and processing of placental material [45].

## 6. Placenta-Related Diseases and Microbiome

Emerging as a possible modulator of pregnancy-related diseases, such as fetal growth restriction (FGR), preeclampsia, gestational diabetes, and premature births, is microbiome dysbiosis [69,86,87,88]. Through microbial DNA or metabolite synthesis, transient microbial communities in the placenta may control the maternal immune response, thereby altering the maternal tolerance of pregnancy, nutrition transfer, and fetal development [89].

### 6.1. FGR

Using 16S sequencing, the reproductive microbiome was investigated (20 FGR and 20 controls) [90] to compare the alpha and beta diversity and identify the taxonomic characteristics related to FGR. The placenta was shown to contain a wide variety of microorganisms, namely, Proteobacteria, Fusobacteria, Firmicutes, and Bacteroidetes. The alpha and beta diversity did not differ substantially according to FGR status. At the taxonomic level, however, the FGR patients showed a much greater incidence of Neisseriaceae, mucosal hemolytic bacteria known to absorb iron-bound host proteins, including hemoglobin. In addition, the increase in anaerobic bacteria, such as *Desulfovibrio*, suggested the development of a hypoxic environment in the FGR placenta. Further investigation of the reproductive microbiome of FGR samples revealed reduced amounts of H_2_O_2_-producing *Bifidobacterium* and *Lactobacillus*, which switch from respiration to fermentation, a less energetic metabolic pathway, when oxygen levels fall. A source-tracking study revealed that the majority of placental microbial content was derived from an oral source as opposed to a gut or vaginal source.

In an additional case–control investigation, He et al. recruited eight FGR and eight control female subjects, from whom fecal samples were collected in their third trimester before delivery [91]. They implicated that the occurrence of FGR was connected with the modification of the gut using metagenomic sequencing and bioinformatic analysis. Twenty gut microbes were significantly different between the two groups (*p* < 0.05), and the correlation analysis revealed that the genus *Roseomonas* and the genus unclassified family Propionibacteriaceae were significantly positively correlated with the maternal BMI before delivery, the placenta weight, and the neonatal birth weight (BW) percentile (all *p* < 0.05), whereas the genus *Marinisporobacter* and the genus *Sphingobacter* were significantly negatively correlated. Utilizing the Kyoto Encyclopedia of Genes and Genomes database and pathway analysis, they determined that the abundance of the nitrogen metabolism route fell substantially (*p* < 0.05) in the FGR group, whereas the abundance of the amoebiasis pathway rose significantly.

In the study of Stupak et al., proteomic and bioinformatic (LC–ESI-MS/MS mass spectrometry) analyses of placental biomass in 18 physiological and 18 FGR pregnancies were conducted [85]. In the FGR group, the results of significantly higher emPAI values of the proteins representative of the following bacteria were detected: *Actinopolyspora erythraea*, *Listeria costaricensis*, *E. coli*, *Methylobacterium*, *Acidobacteria bacterium*, *Bacteroidetes bacterium*, *Paenisporsarcina* sp., *Thiodiazotropha endol oripes*, and *Clostridiales bacterium*. In the control group, on the basis of proteomic data, the following were found statistically more frequently: *Flavobacterial bacterium*, *Aureimonas* sp., and *Bacillus cereus*. Our study showed that placental dysbiosis may be an important factor in the etiology of FGR. The microbiome in the control material may highlight the protective role of selected bacteria, while that in the study group may indicate their pathogenicity.

### 6.2. Preeclampsia

**Figure 2:**
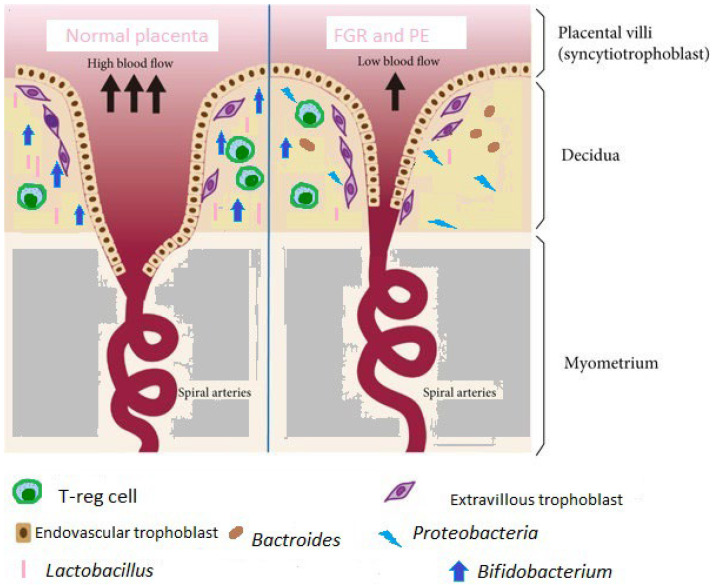
Alterations in the microbiome associated with invasion of the placenta.

In the studies of Wang et al. and Chang et al. on preeclampsia cases compared to healthy controls, a reduction in Firmicutes and an elevation in Bacteroidetes, Proteobacteria, and Actinobacteria were observed [92,93]. A decrease in *Faecalibacterium* and *Akkermansia* and an increase in *Fusobacterium* and *Veillonella* in the PE group were also observed in [94]. Moreover, another study by Lv et al. reported that *Blautia*, *Ruminococcus*, *Bilophila*, *and Fusobacterium* were significantly enriched, while *Akkermansia*, *Dialister*, *Faecalibacterium*, *Gemmiger*, and *Methanobrevibacter* decreased in early-onset PE cases [95]. In these studies, the potential pathological mechanism regulating the placenta’s impaired development leading to PE was explained as a function of a disturbance in the balance of the intestinal microflora and immune homeostasis. The levels of short-chain fatty acids and lipopolysaccharides, as well as the integrity of the nitrate–nitrite–NO pathway, were proposed. These processes led to a disturbance of the trophoblast proliferation and the eventual development of placenta-related syndromes (see Figure 2).

To summarize, in studies on pregnancies with FGR and PE, the placental microbiome differs in the physiological course of pregnancy. 

## 7. Potential Modifying Factors of Gene Transcription

Due to the creation of essential metabolites, such as folate, through DNA synthesis and epigenetic DNA alterations, endogenous bacteria play crucial roles in digestion, immunological function, and even cancer [96]. Two bacterial metabolites, folate and butyrate, can induce epigenetic modifications in neighboring host cells [97,98]. It is possible that microbial components, such as DNA and the metabolic products produced by transitory bacteria, contribute to placental epigenetic alterations. The gut commensals *Lactobacillus acidophilus* and *Bifidobacterium infante* can trigger hypo- and hypermethylation of target genes, which may be implicated in disease risk [99]. Several diseases, including but not limited to fetal growth limitation, have been linked to the differential DNA methylation of certain sites in the placental genome [100]. The inclusion of numerous methylation alterations in placental diseases underlines the importance of DNA methylation in placental development and growth, as well as the absence of a centrally dysregulated mechanism in the initiation of vessel-associated fetal growth restriction [89]. Typically, DNA methylation alterations in the placental tissue are a response to exogenous modulators or stimuli. Nutrition, infection, and general internal illnesses are potential DNA methylation modulators related to prenatal development impairment. More research is required to independently evaluate the association between epigenetic alterations in DNA and microbes.

## 8. Conclusions

The current studies cannot refute the existence of the placental microbiome or its changes. The placenta is a low-biomass organ, and the microbial DNA at this site may represent only remnants of exposure rather than living microorganisms. This is the reason for the inadequate information and classifications of the placenta-associated microbial components (bacterial genetic material) in comparison to the communities located at other mucosal sites [89].

Microorganisms cause the constant development and turnover of the environment of our planet. By offering predictive knowledge of the consequences of disturbances of the microbiome on the lives of other organisms and their surroundings, genomics may hold the secret to curing illnesses and controlling the valuable natural resources and processes that maintain existence on Earth. Currently, a coordinated observation of the implications of disturbances at the most fundamental community levels is required. The findings of the most recent studies are inconclusive regarding the presence of a placental microbiome with low biomass in healthy pregnancies. Oral exposure may have been the source of some of the bacteria that were found in the placenta, or there may have been contamination of the samples. Contamination has a significant influence on the process of determining the likely core bacteria, and further research is necessary to establish the variations in the microbiome in healthy individuals. In many studies, external contamination was excluded, and some researchers pointed out the presence of maternal blood in placental tissue. This problem can be solved by including the maternal sera as a control in the analysis.

In addition, researchers who investigate the effect of bacteria or their components on the development of the immune system and pathologies of pregnancy have emphasized that a great deal of study is required for further elucidation [23]. We are committed to promoting a continuous and open discussion on the methods, pathomechanism, and goals related to reports on the placental microbiome. The practical clinical endpoint is that some investigational microbiome therapeutics (prenatal pro- and prebiotics) may prevent maternal dysbiosis and alter the microbiota transfer to the fetus/newborn [39,64]. However, further research is needed on the type, dosage, and timing of such a treatment.

## Figures and Tables

**Table 1:** Microbiome alteration in particular pregnancy trimesters.

	First Trimester [14,15,19]	Second Trimester [7,20]	Third Trimester [7,20]
Phyla	*Bacteroidetes*, *Firmicutes*	*Proteobacteria*, *Bacteroidetes*, *Actinobacteria*, *Tenericutes*, *Firmicutes*, *Bifidobacteriaceae*, *Enterobacteriaceae*	*Faecalibacterium*, *Actinobacteria*, *Bifidobacterium*, *Enterobacteriaceae*, *Streptococcus*, *Proteobacteria*

**Table 2:** Molecular biology methods used for placental microbiome studies.

Method	Studies
WGS	Aagaard et al. [45]
16S RNA gene sequencing	Amarasekara et al. [43], de Goffu et al. [50], Parnall et al. [49], Aagaard et al. [45], Seferovic et al. [64], Theis et al. [65], Bassols et al. [66], Cahill et al. [67], Collado et al. [68], Jones et al. [69], Leiby et al. [70], Zheng et al. [71,72,73].
16S RNA FISH	Seferovic et al. [64], Cahill et al. [67], Steel et al. [74].
Immunofluorescence	Vanterpool et al. [75].
Metagenomics	De Goffu et al. [50], Aagaard et al. [45], Prince et al. [76], Theis et al. [65], Leiby et al. [70].
Nested PCR	De Goffu et al. [50], Theis et al. [65].
Histology techniques	Lannon et al. [77], Prince et al. [76], Sefrovic et al. [64].
SMURF	Fucks et al. [78].
LC–ESI-MS/MS	Stupak et al. [79].

## Data Availability

No new data were created.

## Abbreviations

BW	birth weight
CSTs	community state types
CS el	elective cesarean section
CS sp	spontaneous cesarean section
FGR	fetal growth restriction
HMP	Human Microbiome Project
IL	interleukin
LPS	endotoxin
OTUs	operational taxonomic units
PlGF	placental growth factor
PRRs	pathogen recognition receptors
sFLT	soluble fms-like tyrosine kinase-1
SMURF	Short Multiple Regions Framework
TLRs	Toll-like receptors
VD	vaginal delivery
WGS	whole genome sequencing
